# Fermented Seeds (“Zgougou”) from Aleppo Pine as a Novel Source of Potentially Probiotic Lactic Acid Bacteria

**DOI:** 10.3390/microorganisms7120709

**Published:** 2019-12-17

**Authors:** Jihen Missaoui, Dalila Saidane, Ridha Mzoughi, Fabio Minervini

**Affiliations:** 1 Laboratory of Analysis, Treatment and Evaluation of Environmental Pollutants and Products, Faculty of Pharmacy, Monastir University, 5000 Monastir, Tunisia; missaouijihen@outlook.fr (J.M.); dalila.saidane@fphm.rnu.tn (D.S.); ridha.mzoughi@rns.tn (R.M.); 2 Dipartimento di Scienze del Suolo, della Pianta e degli Alimenti, Università degli Studi di Bari Aldo Moro, 70126 Bari, Italy

**Keywords:** lactic acid bacteria, *Lactobacillus plantarum*, *Enterococcus faecalis*, *Pinus halepensis* seeds, probiotic traits

## Abstract

Microorganisms inhabiting fermented foods represent the main link between the consumption of this food and human health. Although some fermented food is a reservoir of potentially probiotic microorganisms, several foods are still unexplored. This study aimed at characterizing the probiotic potential of lactic acid bacteria isolated from zgougou, a fermented matrix consisting of a watery mixture of Aleppo pine′s seeds. In vitro methods were used to characterize the safety, survival ability in typical conditions of the gastrointestinal tract, and adherence capacity to surfaces, antimicrobial, and antioxidant activities. Strains belonged to the *Lactobacillus plantarum* group and *Enterococcus faecalis* showed no DNase, hemolytic, and gelatinase activities. In addition, their susceptibility to most of the tested antibiotics, satisfied some of the safety prerequisites for their potential use as probiotics. All the strains tolerated low pH, gastrointestinal enzymes, and bile salts. They displayed a good antibacterial activity and antibiofilm formation against 10 reference bacterial pathogens, especially when used as a cell-free supernatant. Furthermore, the lactic acid bacteria (LAB) strains inhibited the growth of *Aspergillus flavus* and *Aspergillus carbonarius.* Finally, they had good antioxidant activity, although depending on the strain. Overall, the results of this work highlight that zgougou represents an important reservoir of potentially probiotic LAB. Obviously, future studies should be addressed to confirm the health benefits of the LAB strains.

## 1. Introduction

Probiotics are live microorganisms that taken in adequate numbers for a given period of time, benefit human and animal health. To provide benefit to health, probiotic microorganisms have to possess some prerequisites, such as resistance to pH values of the gastrointestinal tract, tolerance to bile salts, and capacity to adhere to intestinal epithelium [1]. 

Many microorganisms involved in food fermentation have been reported as probiotics, such as *Lactobacillus* [1,2], *Bifidobacterium*, *Pediococcus* [1,3], *Lactococcus*, *Propionibacterium* [1,4], *Bacillus* [1], *Enterococcus* [5], and some yeasts [6,7]. Those microorganisms were characterized by, besides the above-mentioned prerequisites, antimicrobial activity and the ability to facilitate the growth of gut beneficial microbes [2,8,9]. Lactic acid bacteria (LAB) are ubiquitous microorganisms isolated from fermented food [10,11], grains [12,13], dairy products [14,15], fruits [16,17], honey-comb [18], and soil [19,20]. Some LAB shows probiotic traits, having effective benefits on crop and livestock production, as well as on human health. In the last decades, many reports have postulated probiotic microorganisms as an adjuvant treatment against gastrointestinal diseases (diarrhea, colon cancer) and skin alterations (photoaging, infections, and cancer) [1,21,22].

*Pinus* sp. is the largest genus of conifers occurring naturally, especially in the north of the Mediterranean area. These conifers are pioneers and expansionist. Leaves, fruits, and seeds of *Pinus* sp. are used worldwide in traditional therapeutic practice and have economic importance because of their richness in secondary metabolites and phytochemicals (e.g., turpentine, terpenes, resins, phenolic compounds, essential oils) showing medicinal and aromatic proprieties [23,24]. *Pinus halepensis* (Aleppo pine) seeds are traditionally used throughout Tunisia and other Arabic countries for preparing a sweet cream pudding called “Assidat-Zgougou”, consumed in Tunisia to celebrate the birth of the Prophet MUHAMMED “sallallahu ’alayhi wa sallam”. However, currently, the consumption of zgougou also occurs during the rest of the year. The first step of Assidat-Zgougou production is the spontaneous fermentation of a watery mixture of ground Aleppo pine seeds. After fermentation, the mixture, called “zgougou” is subjected to coarse filtration to separate the juice from the seed debris. The juice is added with flour and sugar, and the resulting mixture is baked. Alternatively, seeds of *P. halepensis* are used as an aromatic ingredient in ice-cream and candies [25,26]. Secondary metabolites from seeds of *P. halepensis* are used as cosmetics for preventing skin diseases, such as atopic dermatitis, candidiasis, and keratosis, both in healthy and immunocompromised individuals [21]. Anecdotal information is available regarding the benefits to gut from consumption of zgougou. Autochthonous microbes, such as LAB, could be one of the components of zgougou that could benefit human health. However, to our knowledge, nobody thus far isolated and characterized LAB from this spontaneously fermented matrix.

The current study aimed to: (i) Isolate LAB from home-made zgougou; (ii) biotype and identify these LAB; and (iii) explore the potential probiotic capacities of the isolated LAB, as the preliminary basis to exploit them as food supplements (or even medications) for preventing diseases in humans.

## 2. Materials and Methods 

### 2.1. Isolation of Lactic Acid Bacteria (LAB)

Fourteen LAB were isolated from spontaneously fermented zgougou, produced using Aleppo pine’s seeds collected in the Kasserine region (Tunisia) [27]. In detail, zgougou samples (10 mL) were enriched in 90 mL of either de Man Rogosa and Sharpe (MRS) (Oxoid, Basingstoke, Hamsphire, UK) or M17 broth (Oxoid). After incubation, under anaerobic conditions at 37 °C for 24–48 h, 100 µL of each enrichment broth was spread onto MRS or M17 agar media and incubated under the conditions indicated above. Colonies that showed a positive reaction to Gram coloration, a negative reaction to the catalase test, made of non-motile rods or cocci, and capable of acidifying the broth were randomly picked up and subjected to isolation, through at least 2 consecutive streaks. All the isolates were stored at −20 °C in the appropriate broth with 20% glycerol. 

### 2.2. Molecular Biotyping and Identification of LAB 

The bacterial DNA was extracted using the DNeasy^®^ Blood & Tissue Kit (250) (Qiagen, Hilden, Germany) according to the manufacturer’s instructions. The concentration of extracted DNA was estimated by spectrophotometric determination, using the Nanodrop ND-1000 (Thermo Fisher Scientific Inc, Wilmington, USA). The isolates were biotyped by randomly amplified polymorphic DNA (RAPD)-PCR using 3 primers: M13 (5′-GAGGGTGGCGGTTCT-3′) [28], P4 (5′-CCGCAGCGTT-3′) and P7 (5′- AGCAGCGTGG-3′) [29]. The reaction mixture and PCR conditions for primer M13 were according to reference [30,31]. PCR conditions for primers P4 and P7 were those described by reference [29]. PCR products were separated using the MCE-202 MultiNA microchip electrophoretic system (Shimadzu Italia s.r.l., Milan, Italy), using the DNA-2500 IVD Reagent Kit (Shimadzu) and the pGEM^®^ DNA Marker (Promega Italia, Milano, Italy), according to the manufacturer’s instructions. The similarity of the electrophoretic profiles was evaluated by the Pearson product moment correlation coefficient (r) and using the unweighted paired group mathematic average algorithm, through Statistica version 12 for Windows.

Bacterial identification was obtained upon partial sequencing of 16S rRNA gene, using the primers LpigF (5’-TACGGGAGGCAGCAGTAG-3′) and LpigR (5′-CATGGTGTGACGGGCGGT-3′) [32]. *recA* [33] and *pheS* [34] genes were partially sequenced in order to discriminate among species belonging to the *Lactobacillus plantarum* group and *Enterococcus* sp., respectively. PCR products were purified with illustra GFX PCR DNA and gel band purification kit (GE Healthcare Europe GmbH, Milan, Italy) and sequenced at Eurofins Genomics (Ebersberg, Germany). The DNA sequence homology was determined through pair-wise sequence alignments, using BLAST within the NCBI nucleotide collection database.

### 2.3. Probiotic Traits of Lactic Acid Bacteria

#### 2.3.1. DNase and Hemolysis Tests

Ten microliters of bacterial suspension cultured (37 °C, 24 h) in MRS or M17 broth were streaked on DNase-mannitol, and Columbia agar media (Oxoid) supplemented with 5% (*w*/*v*) of defibrinated sheep blood (Thermo Fisher Scientific Inc), in order to test the DNase and hemolytic activities, respectively. Plates were anaerobically incubated for 24/48/72 h at 37 °C [34]. DNase activity was indicated by the appearance of a pink halo around the colonies. *Staphylococcus aureus* ATCC 25923 and *Escherichia coli* ATCC 35218 were used as positive controls for β-hemolytic and α-hemolytic activities, respectively.

#### 2.3.2. Antibiotics Susceptibility 

The strains were subjected to a susceptibility test against Ampicillin (10 µg), Norfloxacin (10 µg), Chloramphenicol (30 µg), Erythromycin (15 µg), Gentamicin (10 µg), Oxytetracyclin (100 µg), Streptomycin (10 µg), Levofloxacin (5 µg), Polymixin B (50 µg), Tetracyclin (30 µg), Ciprofloxacin (5 µg), Nalidixic acid (30 µg), Kanamycin (30 µg), Penicillin G (6 µg), and Rifampicin (30 µg). Bacterial cultures (37 °C, 24 h) in MRS or M17 were appropriately diluted with the respective inoculated broth, in order to adjust their absorbance, read (through an Ultrospec 3000 spectrophotometer, GE Healthcare) at a wavelength of 600 nm, to 0.6 U.A., corresponding to 10^9^ CFU/mL. Bacterial suspensions were inoculated on plates of Muller Hinton (MH) agar (Oxoid) using sterile cotton swabs. After having air-dried plates for 15 min, paper discs containing a single antibiotic were lodged on the plates. After 18 h of incubation at 37 °C, the inhibition zone diameters around each disc were measured. Based on the inhibition zone diameter length, the strains were ranked as resistant (0 mm), intermediate resistant (≤ 8 mm), or susceptible (≥ 10 mm) to antimicrobial substances. Every test was repeated three times. 

#### 2.3.3. Tolerance to Acid, Salt, Pepsin, Pancreatin, and Bile Salt 

Bacterial cultures (37 °C, 18–24 h, under anaerobic conditions), were inoculated (initial absorbance of ca. 0.6 U.A., at a wavelength of 600 nm) in their respective broths (MRS or M17), adjusted at different pH (2.5, 3, 4 and 5), or containing different NaCl concentrations (*w*/*v*) (4, 5, 8 and 12%). After 24 (acid tolerance) and 48 h (salt tolerance) of incubation, bacterial growth was determined spectrophotometrically [35,36]. 

Tolerance to pepsin, pancreatin, and bile salt was assessed using bacterial biomass recovered by centrifugation (10,000× *g*, 5 min, 4 °C; AVANTI-J25, Beckman Coulter) from overnight cultures. Before the tests, biomass was washed twice with phosphate buffer saline (PBS, 130 mM sodium chloride, 10 mM sodium phosphate; pH 7.2) and re-suspended in PBS solution (pH = 2 and pH = 3) containing pepsin (Sigma-Aldrich, St. Louis, MO, USA) (3 mg/mL), or PBS solution (pH = 8) containing pancreatin (Sigma-Aldrich) (1 mg/mL). For assessing the tolerance to bile salt, bacterial biomass was inoculated (initial absorbance of ca. 0.6 U.A., at a wavelength of 600 nm) in MRS or M17 broth with 0.3, 0.5, and 0.8% (*w*/*v*) of ox gall bile (Sigma-Aldrich). In all the cases, cell density of LAB was determined by plate counting on MRS or M17 agar media, soon after inoculation (t = 0 h) and after 1 (pepsin only), 3 (pepsin only), and 4 h of incubation at 37 °C (pancreatin and bile salts) [35]. Before plate counting, the bacterial suspensions were serially diluted with sterile physiological solution and 1 mL of diluted suspensions were inoculated by the pour-plate method. Colonies were counted after 48 h of anaerobic incubation at 37 °C. Survival rates were expressed as follows:

Survival rates% = [cell number (log CFU/mL) after incubation/ cell number (log CFU/mL) after inoculation] × 100(1)

#### 2.3.4. Biofilm Production, Adhesion, and Aggregation Capacities 

The crystal violet method was used to determine the potential of the LAB strains to produce a biofilm [37]. In detail, 100 µL of overnight liquid cultures were added into the microtiter polystyrene plate wells (Thermo Fisher Scientific Inc) previously coated with 100 µL of modified-MRS (2% glucose) or modified-M17 (2% glucose) broths. The cells were allowed to adhere at 37 °C for 24 h. After incubation, the non-adherent cells were removed by washing the wells 3 times with 200 µL of PBS. The adhered cells were stained with crystal violet (Sigma-Aldrich) (100 µL/well, 0.1%, *w*/*v*, solution) for 30 min. Wells were subsequently washed 5 times with PBS to remove the excess stain. After 30 min of incubation at room temperature, the absorbance at 640 nm was determined using a microtiter plate reader (Biochrom Asys Expert Plus microplate reader, UK). Wells containing non-inoculated broth were used as a negative control. Results were expressed by subtracting the absorbance value of this negative control from the absorbance value recorded for each inoculated well. Each experiment was performed in triplicate [38].

Congo Red (CR) (Oxoid) binding assay was used to assess the adhesion capacity of LAB. One hundred microliters of overnight liquid culture were streaked on MRS agar supplemented (after sterilization) with CR (0.01%, *w/v*). After incubation (37 °C for 48–72 h), plates were examined, and the intense red colonies were considered as CR-bound cells [39]. 

The auto-aggregation activity of LAB was assessed using bacterial biomass recovered by centrifugation (5,000× *g*, 15 min, 4 °C) from overnight cultures. Biomass was washed twice end re-suspended in PBS and adjusted to ca. 10^9^ CFU/mL, using spectrophotometric determination. Cell suspensions (4 mL) were mixed vigorously, and then auto-aggregation was checked during 5 h of incubation at ambient temperature. Every hour, the absorbance (at 600 nm) of a mixture of the upper suspension (100 µL) and PBS (3.9 mL) was determined [40]. The auto-aggregation potential was calculated as follows:

AA% = 1 − (At/A0) × 100(2)

where A_t_ represents the absorbance after 1, 2, 3, 4, or 5 h, and A_0_ represents the absorbance at t = 0.

#### 2.3.5. Cell Surface Hydrophobicity Test 

The degree of hydrophobicity of LAB was determined by estimating cellular adhesion to hydrocarbons [36]. Liquid cultures (24 h, 37 °C, anaerobic conditions) of LAB were centrifuged (6000× *g*, 5 min), and the pellets were washed twice and re-suspended in Ringer Solution (6% NaCl, 0.00075% KCl, 0.01% CaCl_2_ and 0.01% NaHCO_3_). The absorbance was measured at 600 nm (reading 1). Then 1.5 mL of the cell suspension was mixed with an equal volume of chloroform (Sigma-Aldrich), ethyl acetate (Sigma-Aldrich), or n-hexadecane (Sigma-Aldrich), and incubated for 30 min at room temperature. After incubation, the absorbance of 1 mL carefully collected from the upper phase of suspension was determined (reading 2). The hydrophobicity activity was calculated using the following equation:

Hydrophobicity% = [[A600 nm (reading 1) − A600 nm (reading 2)]/A600 nm (reading 1)] × 100(3)

#### 2.3.6. Enzyme Activities 

Extracellular amylase, protease, and lipase activities of the LAB strains were qualitatively determined, according to the method described by Maria et al., 2005 [41]. For amylase and protease, soluble starch (Sigma-Aldrich) (2%, final concentration) and gelatin (Sigma-Aldrich) (0.4%, final concentration) was added to MRS or M17 agar, respectively. For the gelatinase test, *S. aureus* ATCC 25923 and *E. coli* ATCC 35218 were used as positive and negative controls, respectively. Extracellular esterase was measured as well, according to the method described by Carrim et al., 2006 [42]. For the lipase and esterase, Tween 20 (Sigma-Aldrich) (1%) and Tween 80 (Sigma-Aldrich) (1%) were added to the peptone agar medium (Himedia, Mumbai, India), respectively. 10 µL of bacterial suspension cultured overnight in MRS or M17 broth were adsorbed on Whatman discs (3 mm diameter). Inoculated discs were lodged on plates containing media with soluble starch, gelatin, Tween 20, or Tween 80. After incubation (37 °C, 24–48 h), plates were examined and the presence of a clear halo around the disc indicated the presence of enzyme activity.

#### 2.3.7. Antimicrobial Activity

The antibacterial activity of LAB strains was tested against the following target microorganisms grown in nutrient broth (NB) (Oxoid) at 37 °C for 24 h: *Staphylococcus aureus* ATCC 25923, *Staphylococcus epidermidis* CIP 106510*, Micrococcus luteus* NCIMB 8166*, Escherichia coli* ATCC 35218, *Listeria monocytogenes* ATCC 19115*, Pseudomonas aeruginosa* ATCC 27853, *Enterococcus faecalis* ATCC 29212, *Salmonella* Typhimurium ATCC 1408*, Bacillus cereus* ATCC 11778, and *Vibrio parahaemolyticus* ATCC 17802. Before the test, the spent broth recovered from 24 h-old LAB cultures was neutralized by 1M NaOH and represented the cell-free supernatant (CFS). Plates of MH agar were singly inoculated, through spread technique, with 100 μL of liquid culture of target microorganisms. The CFS was loaded in wells of 5 mm diameter. After 60 min of incubation at 4 °C, plates were transferred into an incubator set at 37 °C. After 24–48 h of incubation, the antibacterial activity of each CFS was expressed by measuring the diameter of the inhibition zone (clear zone) around the well [43]. Each CFS was tested in triplicate.

The minimum inhibitory concentration (MIC) of the CFS from LAB was evaluated by filling 96 well polystyrene microplates (NUNC^TM^, Denmark) with different dilutions of CFS and MH broth singly inoculated with target microorganisms [43]. The inhibitory effect was rated by measuring the ability of the target microorganism to grow in the presence of the CFS, spectrophotometrically at 600 nm. The test was performed in triplicate. The MIC value was the lowest concentration of CFS that did not allow the growth of the target microorganism. The minimum bactericidal concentration (MBC) was assessed by inoculating, on MH agar, 10 µL from the well containing bacterial suspension that had not grown after microplate incubation. After incubation (37 °C, 24 h), plates were inspected for bacterial growth and MBC was defined as the lowest concentration of CFS showing bactericidal activity.

A dual culture technique was used for determining inhibitory activity of LAB strains against *Aspergillus carbonarius* and *Aspergillus flavus* [44]. Before the assay, fungal conidia were harvested and cultured for 7 days at 28 °C on Potato Dextrose Agar (PDA, Oxoid). Then, the surface of the PDA plate was washed with 5 mL of sterile peptone water (0.1%) (Himedia) mixed with one drop of Tween 80 (Sigma-Aldrich). The resulting mixture of sporangiospores and hyphal fragments was filter-sterilized and transferred to a sterile tube to separate conidia from hyphal fragments [44]. 10 µL of each LAB strain, cultured overnight at 37 °C in MRS or M17 broth, was streaked across the center of MRS or M17 agar plate. 10 µL of fungal suspension of (10^6^ spores/mL) was placed at the side (2.5 cm far) of the LAB streak. One control plate, containing just the fungal inoculum, was also prepared. Plates were then incubated at 37 °C for 7 days. Mycelial growth was measured when mycelium in the control plate was fully developed, and the antifungal activity was expressed as follows:

GI% = (R1 — R2)/R1 × 100(4)

where GI% represents the percentage of fungal growth inhibition, R1 is the farthest radial distance (measured in mm) from the tested LAB streak, reached by the fungal colony, and R2 is the distance from the point of fungal inoculation to the tested LAB streak, namely 2.5 cm. GI was categorized on a scale from 0 to 4, where 0 = no GI, 1 = 1% to 25% GI, 2 = 26% to 50% GI 3 = 51% to 75% GI and 4 = 76% to 100% GI [45,46].

In addition, another method was used for determining the antifungal activity of CFS of each strain [47]. In order to qualitatively evaluate the chemical nature of the possible antifungal compounds present in each CFS, CFS, obtained as described above, was subjected to pH adjustment (pH 6.5 with 46%, *w*/*v*, NaOH aqueous solution) and added (10%, *v*/*v*) to Sabouraud Dextrose (SD) Agar (Oxoid) (pH 4) inside a petri dish (20 mL/plate). The plate was centrally inoculated with 10 μL of the sporal suspension (prepared as described above) and incubated at 25 °C. Control plates containing SD agar and 10% (*v*/*v*) SD broth were also prepared and inoculated with a fungal spore suspension. After a 4 to 7 days incubation period (depending on the required time by each mold to grow and fill the control plate), the area of mycelial growth in both treated (AT) and control (AC) plates was calculated using the averaged diameter, assuming a circular growth. The percentage of growth inhibition (I) was calculated as follows:

I = 100 × (AC − AT/ AC)(5)

Antifungal activity was quantified by broth microdilution assay according to the National Committee for Clinical Laboratory Standards [47,48] with slight modifications. Microdilution assays were performed with a 96-well sterile microtiter plate. The CFS was dispatched, as such or diluted, into each well (180 µL). Then, 2 µL of fungal spore suspension was added at a final concentration of 10^5^ spores/mL. Subsequently, the fungal growth was visually examined after 48 h of incubation at 25 °C. MIC was considered as the highest dilution of the CFS capable of inhibiting fungi [48]. The fungicidal activity of each CFS was evaluated on the suspension containing the dilution level that did not show visible fungal growth. 100 µL were taken from the well and spotted onto the MRS and M17 agar plates. After incubation at 37 ° C for 48 h, plates were examined for fungal growth. The highest dilution of the CFS with fungicidal activity was defined as the minimum fungicidal concentration (MFC).

#### 2.3.8. Inhibition of Biofilm Production

The ability of LAB strain to inhibit biofilm production was estimated according to the method described previously [39]. In detail, the reduction, by metabolically active cells of XTT (2, 3-bis (2-methyloxy-4-nitro-5-sulfophenyl)-2H-tetrazolium-5-carboxanilide) (Sigma-Aldrich), a tetrazolium salt, to a colored formazan was quantified colorimetrically. LAB cells or LAB CFS (absorbance of 0.6 U.A. at 600 nm) were co-inoculated with target pathogenic bacteria (grown in NB, at 37 °C for 18–24 h; absorbance of 0.3 U.A. at 600 nm) in 96-well polystyrene microplates containing brain heart infusion (BHI) (Oxoid) broth with 2% glucose (*w*/*v*). Negative control wells contained just BHI with glucose, whereas positive control wells contained BHI with glucose, inoculated with pathogenic bacteria. XTT solution (1 mg/mL) was prepared in PBS and filtered-sterilized and menadione (Sigma-Aldrich) solution (0.4 mM) was prepared in acetone and filtered-sterilized immediately before each assay. After incubation (24–48 h, 37 °C), the biofilms were firstly washed 5 times with PBS, and then 180 μL PBS and 20 μL of XTT-menadione solution (12.5:1 *v*/*v*) were added to each of the wells. The microplate was then incubated for 3 h in the dark at 37 °C. After incubation, the color change in the suspension was measured through a Multiskan FC Microplate Photometer (Thermo Fisher Scientific Inc) set at a wavelength of 492 nm. 

The percentage of inhibition of biofilm production was calculated using the equation:

[(A growth control − A sample)/A growth control] × 100(6)

where “a growth control” is the absorbance of the well containing the positive control, and “a sample” is the absorbance of the wells containing the tested LAB strain. Each assay was repeated 3 times.

#### 2.3.9. Antioxidant Activity

The antioxidant activity of the LAB strains was assayed through 3 methods: Radical scavenging activity, β-carotene bleaching assay, and reducing power antioxidant activity. Before assays, bacterial strains were prepared as follows. After having cultured (24 h at 37 °C) each LAB strain, bacterial cells were collected by centrifugation (8000× *g*, 10 min, 4 °C). Afterward, the CFS from each bacterial strain was recuperated and filtered by a sterile filter (0.22 µm), whereas the cell pellets were re-suspended in sterile distilled water. For radical scavenging activity, 2,2-DiPhenyl-1-PicrylHydrazyl (DPPH) (Sigma-Aldrich) was used and the test was carried out according to the method of Ali Bougataf et al. [49]. In detail, 167 µL of each test sample were mixed with 167 µL of DPPH (2.5 g/L) and 667 µL of methanol (80%, *v*/*v*). The reaction mixture was incubated in the dark up to 120 min at 25 °C. The absorbance of the reaction mixture was measured at 517 nm at 0, 10, 20, 30, 60, and 120 min. Two positive controls were used: Butyl hydroxytoluene (BHT) (Sigma-Aldrich) (0.45 g/L in methanol 80%) and ascorbic acid (1%). A reaction mixture consisting just of 167 µL of DPPH and 833 µL of methanol (80%) was always inserted as the blank. The radical scavenging activity was calculated as follows:

DPPH radical-scavenging activity (%) = [(A_blank_ – A_sample_) / A_blank_] × 100 (7)

where A_blank_ is the absorbance (at 517 nm) of the reaction mixture containing all reagents without the LAB cells or CFS, and A_sample_ is the absorbance in the presence of the LAB cells or CFS.

The β-carotene bleaching assay was performed according to Trabelsi et al. [50], with slight modifications. In detail, 1 mg of β-carotene (Sigma-Aldrich) was dissolved in 5 mL of chloroform; then 25 mL of linoleic acid (Sigma-Aldrich) and 200 mg of Tween 40 (Sigma-Aldrich) were added. Chloroform was evaporated by treating the sample at 40 °C in a vacuum centrifuge (SpeedVac Concentrator SPD121P, Thermo Scientific). Then, 50 mL of demineralized water was added, and the mixture was vigorously shaken. The emulsion obtained was freshly prepared before each experiment. 2.5 mL of the β -carotene: Linoleic acid emulsion was mixed with an equal volume of the test samples that were prepared in the same way described previously for the DPPH assay. An equal amount of methanol 80% was used for the blank sample. BHT (0.1% in methanol 80%) was used as a positive control. Three replicates were prepared for each of the samples. Readings of all samples were performed spectrophotometrically at 470 nm immediately (t = 0 min), and after 6, 24 and 48 h.

The results were expressed as percentage of antioxidant activity (AA%), using this equation:

AA%= [1 − [(A0 sample-An sample)/(A0 control-An control)]] × 100(8)

where “A_0_ sample” is the absorbance at 470 nm at the start of the reaction, “A_n_ sample” is the absorbance after 6, 24, or 48 h, “A_0_ control” is the absorbance of a mixture not containing LAB (control), and “A_n_ control” is the absorbance of the control after 6, 24, or 48 h.

The reducing power activity of LAB strains was determined according to the method of Sung Ho-son et al. [51]. The mixture, containing 200 µL of LAB cell suspension or CFS, 200 µL of 0.2 M sodium phosphate buffer (pH 6.6) and 200 µL of 1% potassium ferricyanide (Sigma-Aldrich), was incubated at 50 °C for 20 min. Then, 0.2 mL of trichloroacetic acid (10%, *w*/*v*) was added, and the mixture was centrifuged (8000× *g*, 10 min, 4 °C). Then, 500 µL of the supernatant was collected and mixed with 100 µL of 0.1% ferric chloride (Sigma-Aldrich) and 400 µL of distilled water and left to react for 10 min. The absorbance reading of the samples was measured at 700 nm, and L-cysteine (Sigma-Aldrich) was used as a positive control.

### 2.4. Statistical Analyses 

The experimental data obtained were expressed as means ± SD. For all experiments, the differences between the control and treated groups were analyzed by STUDENT TEST, using XLSTAT 2018.

## 3. Results

### 3.1. Safety of Lactic Acid Bacteria (LAB) Strains Identified from Zgougou

**Figure 1:**
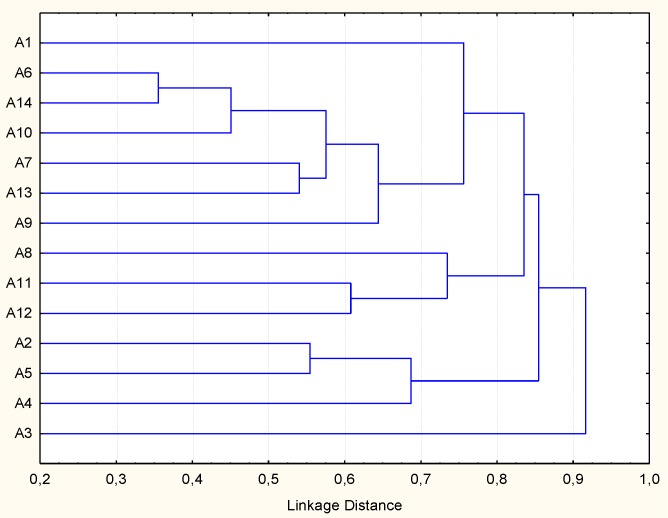
Dendrogram obtained by combined randomly amplified polymorphic DNA-polymerase chain reaction (RAPD-PCR) patterns of the isolates from fermented zgougou. Primers M13, P4, and P7 were used for RAPD-PCR analysis. Cluster analysis was based on the Pearson moment correlation coefficient (r) and unweighted pair grouped method with arithmetic average.

The LAB isolated from zgougou showed different RAPD-PCR profiles (Supplementary Figures S1–S3). The dendrogram built upon the combination of the profiles (Figure 1) showed that the linkage distance between isolates varied from ca. 0.35 (isolates A6 and A14) to ca. 0.90 (isolates A1 and A3). Therefore, all the isolates were subjected to molecular identification. Two strains (A2 and A3, from MRS plates) were allotted to *Lactobacillus plantarum*, one strain (A1, from MRS) to *Lactobacillus paraplantarum* and all the other strains (A4-A14, from M17 plates) to *Enterococcus faecalis*.

No LAB showed DNAse and α- or β-hemolytic activity (data not shown). All the LAB strains were susceptible to chloramphenicol, levofloxacin, penicillin G (except for *E. faecalis* A11), polimixin B sulfate, rifampicin and tetracyclin (Table S1). Resistance to kanamycin and oxytetracyclin was found for almost all the strains. The remaining 7 antibiotics showed different activity depending on the species and strain. Strains of the *L. plantarum* group were resistant to streptomycin, norfloxacin, and ciprofloxacin, whereas all the enterococci strains were inhibited by these three anitbiotics. *E. faecalis* A6, A7, A10, A12, and A14 were susceptible to 13 out of 15 antibiotics. 

### 3.2. Tolerance to Stressing Environmental Conditions 

**Figure 2:**
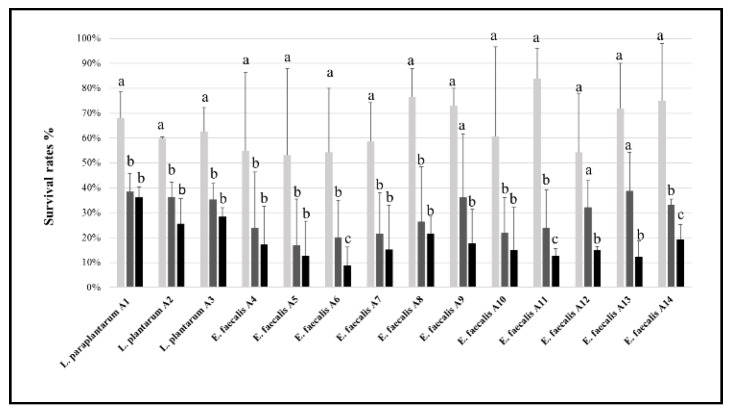
Survival rates (%) of the lactic acid bacteria (LAB) strains grown at 37 °C for 48 h in MRS or M17 media containing NaCl, at a concentration of 4% (light grey bars), 8% (dark grey bars), or 12% (black bars). For each strain, the bars sharing one common letter (a–c) indicate not significantly (*p* > 0.05) different values of survival rate.

All the LAB strains showed good tolerance to the lowest NaCl concentration, their survival rate ranging from ca. 54% (*E. faecalis* A4, A5, A6, and A12) to 84% (*E. faecalis* A11) (Figure 2). Overall, all of them were susceptible to NaCl concentrations of 8% and 12%. However, among them, *L. paraplantarum* A1 showed the highest survival rates (37%) even after exposure to relatively high NaCl concentrations.

**Figure 3:**
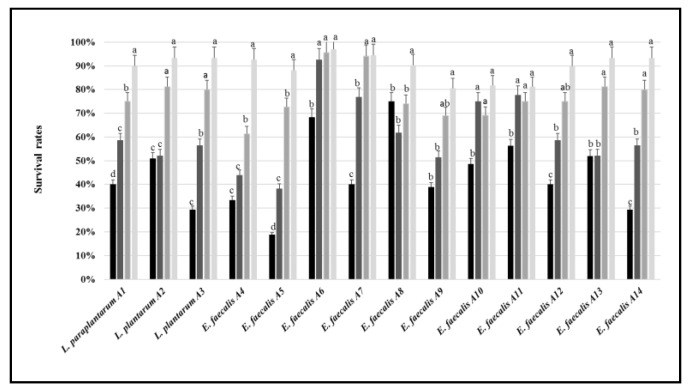
Survival rates (%) of the LAB strains grown at 37 °C for 24 h in MRS or M17 media adjusted at pH 2.5 (black bars), 3 (dark grey), 4 (grey), or 5 (light grey). For each strain, the bars sharing one common letter (a–d) indicate not significantly (*p* > 0.05) different values of survival rate.

All the selected strains exhibited high tolerance after 24 h of incubation in growth media adjusted at pH 4 or 5, the survival rate ranging from 60% (*E. faecalis* A4) to 96% (*E. faecalis* A6) (Figure 3). LAB grown at pH 3 showed different survival rates depending on the strain. For instance, within strains of *E. faecalis*, survival rate ranged from ca. 40% (A5) to 90% (A6). Despite being grown at pH 2.5, *L. plantarum* A2, and *E. faecalis* A6, A8, A11, and A13 showed relatively high survival rates (from approximately 50% to 75%).

The tested LAB showed different tolerance to pepsin after 1–3 h of exposure (Table S2). The strains belonging to the *L. plantarum* group (A1, A2, A3) and the strains A4, A7, and A14, allotted to *E. faecalis*, were the most tolerant after 3 h of treatment with pepsin at pH 2, with survival rates ranging from 60% (*E. faecalis* A7) to 77% (*L. paraplantarum* A1). After 3 h of exposure with pepsin at pH 3, all the strains were more tolerant than in the treatment at lower pH, showing survival rates between ca. 48% (*E. faecalis* A5 and A11) and 79% (*E. faecalis* A8). Overall, strains of *E. faecalis* showed higher survival rates after 4 h exposure to pancreatin, compared to lactobacilli strains (Table S2).

**Figure 4:**
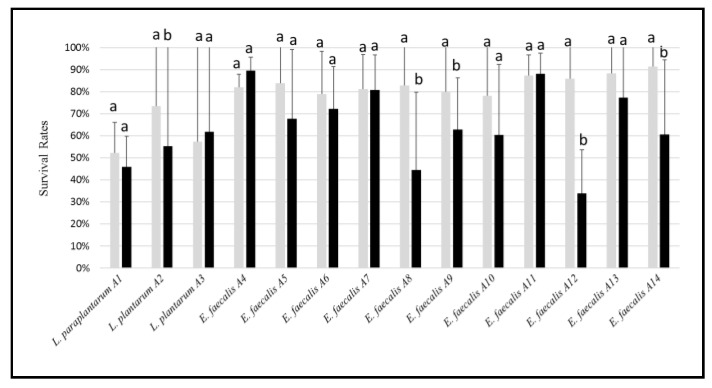
Survival rates (%) of the LAB strains exposed for 0 (light grey bars), and 4 (black bars) h to 1% bile salt. For each strain, the bars sharing one common letter (a–b) indicate not significantly (*p* > 0.05) different values of survival rate.

All the LAB strains could tolerate the exposure to 1% bile salts A5 (Figure 4), being *E. faecalis* A4 and A11 the two most tolerant strains, showing survival rates of ca. 90%. *E. faecalis* A12 was the most susceptible strain to bile salts (survival rate of ca. 30%). Similar results were found when LAB strains were exposed to lower concentrations of bile salts (data not shown).

### 3.3. Biofilm Production, Adhesion and Aggregation Capacities

**Figure 5:**
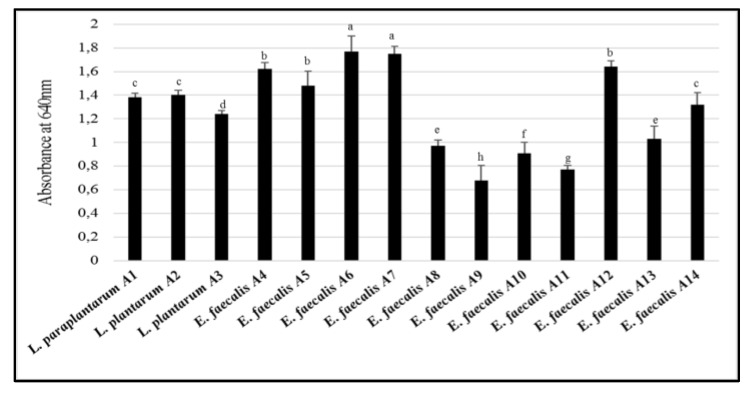
The capacity of producing biofilm by the LAB strains, expressed in terms of absorbance at 640 nm of crystal violet. The bars sharing one common letter (a–h) indicate not significantly (*p* > 0.05) different values of biofilm production capacity.

*E. faecalis* A6 and A7 were characterized by the highest capacity to produce biofilm (Figure 5). The lowest capacity (absorbance values lower than 1) was found for *E. faecalis* A8, A9, A10, and A11, whereas the remaining strains had the intermediate capacity. *L. paraplantarum* A1, *L. plantarum* A2, *E. faecalis* A4, A5, A6, A7, A12, A13, and A14 showed high adhesion capacity (data not shown). All the strains had a very high capacity of auto-aggregation, ranging from 92% to 98% (data not shown).

### 3.4. Cell Surface Hydrophobicity Test

The hydrophobicity, tested using n-hexadecane, ranged between ca. 55% (*E. faecalis* A6) and 90% (*L. paraplantarum* A1, *L. plantarum* A2, *E. faecalis* A12 and A13), whereas when chloroform was used in the test, the hydrophobicity varied from ca. 46% (*E. faecalis* A10) to 73% (*E. faecalis* A5 and A9) (Table 1). Overall, lower values of hydrophobicity were found when ethyl acetate was used, ranging between ca. 8% (*E. faecalis* A9) and 66% (*L. plantarum* A2 and *E. faecalis* A13). All the strains were regarded as highly hydrophobic, at least towards one of the three compounds used in testing. The only exception was for *E. faecalis* A4, which was ranked as having intermediate hydrophobicity.

### 3.5. Enzyme Activities

None of the tested strains exerted gelatinase activity. *E. faecalis* A5, A7, A9, A11, A12, and A13 showed the highest lipase activity, with a halo diameter longer than 10 mm (data not shown). All the other strains had lower lipase activity (halo diameter ranging from 4 to 10 mm). High esterase activity was found for four strains (*E. faecalis* A4, A8, A11, and A14). The remaining strains showed lower levels of esterase. All the strains displayed high amylase activity, with the exception of *L. plantarum* A2 and A3, and *E. faecalis* A4 (data not shown).

### 3.6. Antibacterial Activity

All the LAB strains inhibited at least 7 out of 10 target bacteria (Table S3). Six strains (*E. faecalis* A5, A7, A8, A9, A10, and A12) inhibited all the target bacteria. The pathogenic indicator *E. faecalis* ATCC 29212 was inhibited by all the LAB strains, including those belonging to the same species. *M. luteus* and *E. coli* were especially inhibited by the strains of the *L. plantarum* group. *S.* Typhimurium, *B. cereus,* and *V. parahaemolyticus* could hardly be inhibited by only some of the tested LAB. MIC values of CFS of the tested LAB ranged between ca. 2.5 × 10^8^ and 4.5 × 10^8^ CFU/mL. Bactericidal activity of the CFS ranged from 2.5 × 10^8^ to 5.0 × 10^8^ CFU/mL. All the CFS from strains belonging to *E. faecalis* had no bactericidal activity towards all the target bacteria, with the exception of *S. aureus*, *S. epidermidis*, *M. luteus*, and *E. faecalis* (data not shown).

The LAB strains could inhibit biofilm formation by the pathogenic bacteria, with a percentage ranging from ca. 31% to 89% (Table S4). Among the most active strains, *E. faecalis* A5 showed inhibitory activity against biofilm formed by *S. epidermidis*, *S. aureus*, *M. luteus, P. aeruginosa, or E. faecalis*. Biofilm production by *S. aureus* was strongly inhibited by *E. faecalis* A12. *E. faecalis* A6 and A7 were the most active biofilm inhibitors against *P. aeruginosa* and *E. coli*, respectively. *E. faecalis* A4 and A13 strongly inhibited biofilm formation by the pathogenic bacteria *E. faecalis* and *S.* Typhimurium, respectively.

### 3.7. Antifungal Activity

The LAB strains displayed antagonistic activity against *Aspergillus flavus* and *Aspergillus carbonarius* (Table 2)*. A. flavus* was inhibited by the cell pellets, with values ranging from ca. 50% (*L. paraplantarum* A1 and *L. plantarum* A3) to 71% (*E. faecalis* A13). The inhibitory activity of the cells of LAB strains against *A. carbonarius* varied from ca. 59% (*E. faecalis* A14) to 97% (*L. paraplantarum* A1). The results obtained using the CFS from LAB were in good agreement with those obtained using cells. However, the CFS from the strains A1, A2 and A3, belonging to the *L. plantarum* group, were characterized by the lowest inhibitory activity against both fungal species. Overall, *A. carbonarius* was more sensitive to LAB cells or CFS than *A. flavus*. MIC values varied from 40% (*L. plantarum* A2) to 80% (*E. faecalis* A12), and from 50% (strains of the *L. plantarum* group) to 70% (*E. faecalis* A8, A9, A10, and A11) against *A. flavus* and *A. carbonarius*, respectively (data not shown). Results on MFC showed that the LAB strains produced compounds that inactivated the two target fungi.

### 3.8. Antioxidant Activity

**Figure 6:**
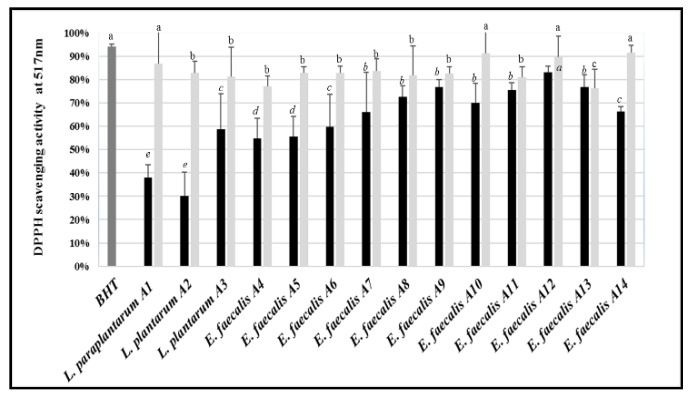
2,2-DiPhenyl-1-PicrylHydrazyl (DPPH) radical scavenging activity (%) of the LAB strains tested as whole cultures (black bars) or CFS (light grey bars). Butyl hydroxytoluene (BHT) was used as a positive control. The bars sharing one common letter (a–e) indicate not significantly (*p* > 0.05) different values of radical scavenging activity.

All the tested LAB, both as bacterial culture and CFS, displayed lower radical scavenging activity than the control (BHT) (Figure 6). When the whole bacterial cultures were tested, the scavenging activity against DPPH ranged between ca. 30% (*L. plantarum* A2) and 83% (*E. faecalis* A12). Compared to bacterial cultures, the CFS showed higher antioxidant activity, with values ranging from ca. 76% (*E. faecalis* A13) and 90% (*E. faecalis* A10 and A12).

**Figure 7:**
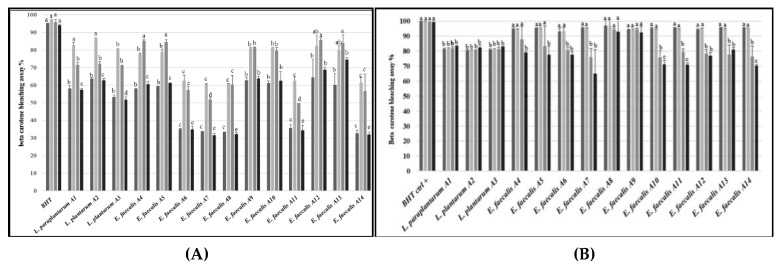
Antioxidant activity (%) of the LAB strains, tested as whole cultures (**A**) or CFS (**B**), determined after 0 (dark grey), 6 (light grey), 24 (grey), or 48 h (black bars), using the β-carotene bleaching assay. BHT was used as a positive control. The bars sharing one common letter (a–e) indicate not significantly (*p* > 0.05) different values of antioxidant activity.

When the β-carotene bleaching assay was used, bacterial cultures of the tested LAB strains showed lower antioxidant activity (varying from ca. 30% to 65%) than the positive control at the beginning of incubation (Figure 7A). The antioxidant activity of the LAB cultures increased after 6 h of incubation. A further increase was found at 24 h just for *E. faecalis* A4 and A5, whereas the activity of the other strains decreased or remained constant. Compared to 6 or 24 h of incubation, all the bacterial cultures showed lower antioxidant activity at 48 h. At this incubation time, the most active strains were *E. faecalis* A9, A10, A12, and A13. Overall, CFS showed higher antioxidant activity than the corresponding bacterial cultures, although lower than the control (Figure 7B). In detail, the strains belonging to the *L. plantarum* group (A1, A2, and A3) showed quite a constant antioxidant activity, regardless of the incubation time. Strains of *E. faecalis* showed higher activity than those of the *L. plantarum* group after 0 and 6 h of incubation. When incubation was prolonged to 48 h, the antioxidant activity decreased for all the strains of *E. faecalis*, except for the strains A8 and A9, whose activity was constant.

**Figure 8:**
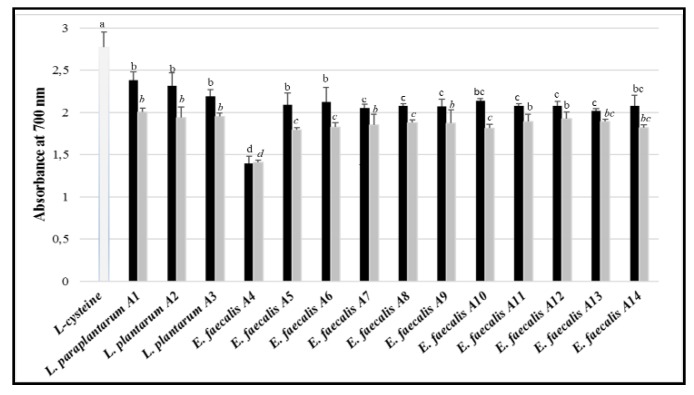
Antioxidant activity, expressed as absorbance at 700 nm, of the LAB strains, tested as whole cultures (black) or CFS (grey bars), using the FRAP assay. l-cysteine was used as a positive control. The bars sharing one common letter (a–d) indicate not significantly (*p* > 0.05) different values of antioxidant activity.

Antioxidant activity was also tested using the ferric reducing antioxidant power (FRAP method. Overall, whole bacterial cultures showed higher activity than the CFS. The three strain of the *L. plantarum* group had higher activity than those belonging to *E. faecalis* (Figure 8). When CFS was used, lower differences in antioxidant activity were found among the tested LAB strains, except for *E. faecalis* A4, which showed the lowest antioxidant potential.

## 4. Discussion

During human history, naturally fermented foods and beverages have been an important part of the diet. The majority of fermented food items are still produced using traditional protocols [52]. Although few issues have been related to the consumption of specific fermented foods, in most of cases they seem to benefit human health [52,53]. Microorganisms inhabiting fermented foods, either as naturally present or added as starters, represent the main link between consumption of these food and health benefits. Their importance dates back to the studies by Elie Metchnikoff about the correlations between consumption of fermented milks and longevity of individuals living in some Bulgarian regions [54]. Microbial consortia of fermented foods lead to the improvement of sensory, shelf-life, nutritional, and functional quality [53]. The potential probiotic activity of several fermentative bacteria (e.g., lactic acid bacteria, LAB) living in fermented foods has been reported. *Lactobacillus* and *Enterococcus* are among the most commonly used genera of probiotics [55]. Although most of applications of probiotic microorganisms for benefiting human health regard gastrointestinal and immune system functions, in the last decade the use of probiotics for preventing or even treating skin diseases (e.g., atopic dermatitis, actinic keratosis, fungal and bacterial infections) is attracting the attention of an increasing number of researchers [22,56,57,58,59].

In the current study, the probiotic potential of 14 LAB isolated from zgougou, a fermented food based on Aleppo pine’s seeds was assessed, as recommended by the FAO/WHO (2002). The screening was performed using in vitro tests, which represent the first step before starting the in vivo evaluation of health benefits. The strains belonging to the *L. plantarum* group or *E. faecalis,* showed no DNase, hemolytic, and gelatinase activities. The latter enzyme could be harmful in the case of the application of probiotic microorganisms to host districts, such as skin and gut [60,61]. In contrast with our results, production of gelatinase by pathogenic strains of *E. faecalis* has been previously observed [62,63,64].

The presence of genetic determinants of antibiotic resistance represents another issue, since they could be horizontally transferred to bacteria inhabiting the human gastrointestinal tract [65,66,67,68]. The LAB strains from zgougou were susceptible to chloramphenicol and tetracyclin. The gene transfer of resistance towards these two antibiotics is emerging for lactobacilli [69,70,71]. In addition, all the strains considered in this study were inhibited by three other antibiotics. In spite of the reported resistance to polimixin B by enterococcal strains [72], all the strains of *E. faecalis* tested in this study were inhibited by this antibiotic. Almost all the strains showed resistance to kanamycin and oxytetracyclin. A pattern of specific antibiotic resistance was recognized for strains the *L. plantarum* group, but not for strains of enterococci. In detail, lactobacilli resisted to five more antibiotics, besides kanamycin and oxytetracyclin. These results roughly confirm previous studies about antibiotic resistance in LAB [35,72,73,74]. However, the absence of antibiotic resistance determinants has been recently resulted from comparative genomic analysis of 50 strains of the *L. plantarum* group of various origin [75]. In addition, one previous report showed that 38 out of 54 strains of *E. faecalis* were susceptible to oxytetracyclin [72]. The absence of DNase and hemolytic activity and the susceptibility to most of the tested antibiotics satisfied some of the safety prerequisites [76,77,78] for the potential use of the LAB isolated from zgougou as probiotic bacteria.

Resistance to low pH, bile salts, pepsin, and pancreatin and, just for eventual use as dietary supplements in aquaculture, high concentrations of NaCl are among the most important selection criteria for probiotics. The survival of potentially probiotic strains in the gastrointestinal juice and stomach acidity is a key functional assessment for their activity in the host [79,80,81,82]. The ability to survive stressing conditions that mimic the gastrointestinal digestion varied depending on the strains considered in this study, although enterococci showed higher resistance to pancreatin than lactobacilli. In addition, 7 out of 10 enterococci strains showed higher tolerance to bile salts, compared to lactobacilli. Similar results were previously reported for bacteria belonging to the same species tested in this study [35,51,68,79,81,83].

The capacity of probiotic microorganisms to adhere to cell surface, produce biofilm, and auto-aggregate plays a pivotal role for colonization of host epithelia (e.g., intestinal, cutaneous), a pre-requisite to help host defense mechanisms against gut and skin infections [37,59]. The results obtained in this study showed that almost all the LAB strains displayed a very high performance of cell adhesion, auto-aggregation, and biofilm formation. Similar results were previously reported for *Lactobacillus* and *Enterococcus* species upon in vitro tests using polysterene, gut epithelial cells, or mucin [35,68,73,84,85,86,87,88]. Hydrophobicity often mediates and strengthens the surface adhesion ability of probiotic microorganisms. Indeed, some components of the microbial cell surface (e.g., glycoproteins, polysaccharides) show strong affinity to hydrophobic compounds [73,87,88,89,90,91]. In this study, most of the tested LAB were highly hydrophobic to n-hexadecane (non-polar solvent) and chloroform (a mono-polar and acidic solvent), whereas they showed lower affinity towards ethyl acetate (a mono-polar basic solvent). Analogous results were reported previously. For instance, *Lactobacillus brevis* had an average hydrophobicity towards chloroform and n-hexadecane, but very low hydrophobicity towards ethyl acetate. *L. plantarum* and *L. paraplantarum* showed high hydrophobic potential towards *n*-hexadecane, besides other non-polar solvents, such as toulene and xylene [35,86,92]. In addition, hydrophobicity of *E. faecalis* towards xylene and hexane had been previously shown [93,94].

Cultivation of microorganisms for production and extraction of hydrolytic enzymes (lipase, amylase and esterase) is a currently valuable biotechnological solution for supplying enzymes for various uses [60,61,95,96,97,98,99,100]. The LAB strains tested in this study showed interesting, but strain-depending variable, enzyme activities, in accordance with previous studies on various species of lactobacilli. Those enzymes can help digestive process in animals and humans [101,102,103].

The ability to inhibit or inactivate undesired bacteria is one of the most attractive traits of potentially probiotic microorganisms. Antimicrobial activity of probiotics may play very important role in modulating gastrointestinal and cutaneous dysbiosis and related diseases [8,104,105,106,107,108]. In this study, the 14 LAB strains had strong growth-inhibiting attributes against at least 7 out of 10 target bacteria, responsible of either food-born disease (e.g., *Listeria monocytogenes, Escherichia coli, Salmonella* Typhimurium, *Bacillus cereus*) or skin infections (e.g., *Micrococcus luteus*, *Staphylococcus aureus*, *Staphylococcus epidermidis*). The LAB strains did not show bactericidal activity, but most of them could control over biofilm formation by the pathogenic bacteria. In agreement with our results, *Lactobacillus* sp. could inhibit the bacterial growth, and had anti-biofilm activity against *L. monocytogenes, E. coli, S.* Typhimurium, *B. cereus*, and *S. aureus* [86,109]. *L. brevis* showed a broad inhibitory spectrum against the same target bacteria used in this study, with the exception of *M. luteus* and *Vibrio parahaemolyticus* [35]. Antibacterial activity has been reported for enterococci too. *E. faecalis* inhibited the growth of *S. aureus, E. faecalis*, and *E. coli* [110]. *E. durans* and *E. faecalis* counteracted proliferation of *L. monocytogenes, E. faecalis, E. coli, S.* Typhimurium, *Pseudomonas aeruginosa*, *B. cereus*, and *S. aureus* [111]. Growth and biofilm formation by *V. parahaemolyticus* were inhibited by almost all the strains used in this study, in agreement with previous studies focused on *Vibrio* sp. [112,113,114].

The LAB strains isolated from zgougou were tested against two fungal species, *Aspergillus carbonarius* and *Aspergillus flavus*, causing diseases or metabolic disorders in gastrointestinal tract and skin. Aspergillosis is correlated to infections by the above-mentioned fungal species in immunocompromised individuals, including burn victims, neonates, individuals with cancer, and HIV infected populations [115,116,117,118]. Some studies suggested the existence of a correlation between aspergillosis and cancer development [119,120,121]. In this study, as assessed through two different assays, the two target fungi were sensitive, with different, strain-dependent degrees, to all the tested LAB and their CFS. In particular, CFS from strains of *E. faecalis* had the highest antifungal activity. This activity could be probably due to the negative influence of some compounds (e.g., organic acids) contained in CFS on fungal proliferation. Strains of *E. faecium* and *E. faecalis* were identified as potential bio-preservative agents to control over pathogenic molds in cereals, fermented foods, and dairy products [122,123]. In addition, CFS extracted from *Lactobacillus* sp. could reduce both the growth and toxin production of *Aspergillus* sp. [124,125,126,127,128,129,130].

Antioxidant activity represents an additional interesting trait in the selection pathway of candidate probiotic microorganisms. Oxidative stress, mainly caused by free radicals, contributes to carcinogenesis and ageing. Antioxidants may counteract the degenerative action triggered on the host by free radicals, thus provoking health benefits. Some antioxidant compounds produced by LAB act as scavengers of reactive oxygen species (ROS) and metal chelators [131,132]. The results obtained in this study showed that the LAB strains isolated from zgougou, especially used in the form of CFS, had good DPPH radical scavenging capacity, possibly due to their extracellular metabolites. The antioxidant activity depended on the strain. Their activity was also confirmed through the FRAP and *β*-carotene bleaching assays. The latter simulates the peroxidation of membrane lipid components (e.g., linoleic acid) [133]. In addition, using the *β*-carotene bleaching assay, CFS showed higher antioxidant activity than whole bacterial cultures. It may be hypothesized that CFS are rich in secondary metabolites and exopolysaccharides (EPS) displaying this biological activity [134]. EPS from *L. plantarum* and *E. faecium* could possess an important, dose-dependent radical scavenging activity [132,135]. In agreement with the results of this study, the antioxidant activity of *Lactobacillus* sp. [50,131,132,134,136,137,138,139] and *Enterococcus* sp. [140,141] decreased the accumulation of ROS during food fermentation. In addition, the lyophilized CFS of *E. faecium* disclosed high antioxidant activity [142] and strains of *Enterococcus* sp. displayed an excellent radical scavenging capacity, thanks to antioxidant compounds present in their supernatant [142].

Zgougou is one of the several fermented foods that thus far has received scarce attention, probably because it is mostly home-made. However, the scarce information does not necessarily mean that it cannot display beneficial, evidence-based effects [53]. Indeed, the results of this work highlight that zgougou represents an important reservoir of potentially probiotic LAB. Obviously, future studies should be addressed to confirm the health benefits of the LAB strains.

## 5. Conclusions

In this study, LAB strains were isolated for the first time from zgougou, a traditional Tunisian fermented food, and characterized for their potential probiotic traits. Interestingly, most of LAB strains belonged to *E. faecalis*; however, differently from most of the strains belonging to the same species but isolated from animal matrices, they did not show any particular pathogenic trait. Although there was no unique LAB strain, possessing at the highest level all the desirable probiotic traits, further studies could allow the assessment of the safe use of these LAB as: (i) Probiotic components tailored to prevention of gastrointestinal and skin diseases (through anti-inflammatory, anti-carcinogenic effects); (ii) starters for food fermentation; and/or (iii) food bio-preservatives.

## Figures and Tables

**Table 1:** Percentage * of cell surface hydrophobicity of the LAB strains towards n-hexadecane, chloroform, or ethyl acetate.

LAB Strain	n-Hexadecane	Chloroform	Ethyl Acetate
*L. paraplantarum* A1	90 ± 0.4 ^a^ (H^§^)	53 ± 0.2 ^bc^ (M^§^)	64 ± 0.2 ^a^ (M)
*L. plantarum* A2	90 ± 0.4 ^a^ (H)	70 ± 0.5 ^a^ (H)	66± 0.0 ^a^ (M)
*L. plantarum* A3	79 ± 0.5 ^b^ (H)	70 ± 1.2 ^a^ (H)	24 ± 2.1 ^c^ (M)
*E. faecalis* A4	64 ± 1.2 ^b^ (M)	66 ± 1.2 ^ab^ (M)	39 ± 0.6 ^bc^ (M)
*E. faecalis* A5	55 ± 1.3 ^c^ (M)	73 ± 0.6 ^a^ (H)	51 ± 1.2 ^a^ (M)
*E. faecalis* A6	75 ± 0.6 ^b^ (H)	65 ± 0.9 ^ab^ (M)	35 ± 0.3 ^bc^ (L§)
*E. faecalis* A7	83 ± 0.5 ^ab^ (H)	71 ± 0.8 ^a^ (H)	21 ± 2.7 ^c^ (L)
*E. faecalis* A8	78 ± 1.2 ^b^ (H)	61 ± 0.3 ^b^ (M)	24 ± 0.6 ^c^ (L)
*E. faecalis* A9	75 ± 0.9 ^b^ (H)	73 ± 0.7 ^a^ (H)	8 ± 0.8 ^d^ (L)
*E. faecalis* A10	85 ± 0.3 ^ab^ (H)	46 ± 0.2 ^c^ (M)	22 ± 0.4 ^c^ (L)
*E. faecalis* A11	77 ± 0.5 ^b^ (H)	59 ± 1.0 ^bc^ (M)	46 ± 0.4 ^b^ (M)
*E. faecalis* A12	90 ± 0.4 ^a^ (H)	53 ± 0.2 ^bc^ (M)	64 ± 0.2 ^a^ (M)
*E. faecalis* A13	90 ± 0.4 ^a^ (H)	71 ± 0.5 ^a^ (H)	66 ± 0.0 ^a^ (M)
*E. faecalis* A14	79 ± 0.5 ^b^ (H)	70 ± 1.2 ^a^ (H)	24 ± 2.1 ^c^ (M)

* Values in the same column with at least one common letter (a–d) showed no significant (*p* > 0.05) differences. H^§^, highly hydrophobic (71–100%); M, intermediately hydrophobic (36–70%); L, lowly hydrophobic (0–35%), according to Thalpa N et al. 2004 [36].

**Table 2:** Antifungal activity * of LAB strains expressed as percentage of mycelial growth inhibition (GI%) caused by the cells or as percentage of inhibition (I%) caused by the cell-free supernatant (CFS).

LAB Strain	*Aspergillus flavus*		*Aspergillus carbonarius*		I% of *A. flavus*	I% of *A. carbonarius*
	GI%	GI Category	GI%	GI Category		
A1	50 ± 3.5 ^b^	2	97 ± 0.6 ^a^	4	40 ± 1.5 ^b^	67 ± 2.0 ^b^
A2	52 ± 3.4 ^b^	3	85 ± 0.8 ^a^	4	28 ± 1.7 ^b^	80 ± 1.0 ^b^
A3	50 ± 3.5 ^b^	3	90 ± 0.6 ^a^	4	25 ± 2.0 ^b^	78 ±1.3 ^b^
A4	56 ± 2.6 ^b^	3	89 ± 1.4 ^a^	4	61 ± 1.1 ^a^	94 ± 2.9 ^a^
A5	63 ± 2.1 ^ab^	3	89 ± 1.3 ^a^	4	59 ± 1.4 ^a^	95 ± 1.1 ^a^
A6	56 ± 2.5 ^b^	3	89 ± 1.3 ^a^	4	58 ± 1.0 ^a^	95 ± 1.1 ^a^
A7	62 ± 1.9 ^ab^	3	87 ± 1.0 ^a^	4	58 ± 9.4 ^a^	95 ± 1.4 ^a^
A8	68 ± 1.7 ^a^	3	77 ± 2.0 ^abc^	4	57 ± 1.1 ^a^	95 ± 1.6 ^a^
A9	66 ± 2.0 ^a^	3	81 ± 1.6 ^a^	4	62 ± 1.2 ^a^	94 ± 1.3 ^a^
A10	70 ± 1.7 ^a^	3	86 ± 1.4 ^a^	4	58 ± 1.1 ^a^	95 ± 1.1 ^a^
A11	70 ± 1.7 ^a^	3	87 ± 1.3 ^a^	4	56 ± 1.2 ^a^	95 ± 1.1 ^a^
A12	53 ± 2.9 ^b^	3	71 ± 2.3 ^bc^	3	54 ± 1.1 ^a^	95 ±0,9 ^a^
A13	71 ± 1.6 ^a^	3	83 ± 1.4 ^a^	4	51 ± 9.7 ^a^	94 ± 1.2 ^a^
A14	68 ± 1.8 ^a^	3	59 ± 2.5 ^c^	3	54 ± 1.1 ^a^	96 ± 1.0 ^a^

* Values in the same column with at least one common letter (a–c) showed no significant (*p* > 0.05) differences.

## Supplementary Materials

The following are available online at https://www.mdpi.com/2076-2607/7/12/709/s1.
