# Supplementary material for: Fermented Seeds (“Zgougou”) from Aleppo Pine as a Novel Source of Potentially Probiotic Lactic Acid Bacteria
Source: Microorganisms. 2019 Dec 17;7(12):709. doi: 10.3390/microorganisms7120709 (PMC6958562; doi:10.3390/microorganisms7120709)
Supplement: Supplementary file 1 [file microorganisms-07-00709-s001.pdf]

**Table S1.** Diameter (in mm) of inhibition zone found for the LAB strains tested with 15 antibiotics**.**

| | A1 | A2 | A3 | A4 | A5 | A6 | A7 | A8 | A9 | A10 | A11 | A12 | A13 | A14 |
|---|---|---|---|---|---|---|---|---|---|---|---|---|---|---|
| ***Inhibitors of cell wall synthesis*** | | | | | | | | | | | | | | |
| *Ampicillin* | 29(S*) | 30(S) | 30(S) | 35(S) | 34(S) | 35(S) | 30(S) | 0 (R*) | 0 (R) | 36 (S) | 0 (R) | 34 (S) | 0 (R) | 0 (R) |
| *Penicillin G* | 30(S) | 38(S) | 32(S) | 36 (S) | 35 (S) | 36 (S) | 35 (S) | 36 (S) | 38 (S) | 34 (S) | 11(R) | 26 (S) | 34 (S) | 8 (I*) |
| *Polymixin B sulfate* | 12(S) | 9(S) | 11(S) | 18(S) | 17 (S) | 15.5 (S) | 18 (S) | 18 (S) | 17 (S) | 16 (S) | 12(S) | 18(S) | 17 (S) | 17(S) |
| ***Inhibitors of proteins synthesis*** | | | | | | | | | | | | | | |
| *Chloromphenicol* | 27(S) | 32(S) | 37(S) | 35(S) | 26(S) | 32(S) | 30(S) | 34(S) | 31(S) | 32(S) | 30(S) | 30(S) | 32(S) | 32(S) |
| *Erythromycin* | 20(S) | 22(S) | 0 (R) | 22(S) | 12 (S) | 12 (S) | 12 (S) | 8(I) | 8(I) | 26(S) | 18(S) | 0 (R) | 12(S) | 10(S) |
| *Gentamicin* | 0 (R) | 8(I) | 8(I) | 8(I) | 8(I) | 10(S) | 8(I) | 24 (S) | 22 (S) | 11(S) | 24 (S) | 8(I) | 18(S) | 23 (S) |
| *Kanamycin* | 0 (R) | 0 (R) | 0 (R) | 0 (R) | 0 (R) | 0 (R) | 0 (R) | 0 (R) | 0 (R) | 0 (R) | 26 (S) | 31 (S) | 0 (R) | 26 (S) |
| *Oxytetracyclin* | 0 (R) | 0 (R) | 0 (R) | 0 (R) | 0 (R) | 0 (R) | 0 (R) | 0 (R) | 0 (R) | 0 (R) | 0 (R) | 0 (R) | 0 (R) | 0 (R) |
| *Streptomycin* | 0 (R) | 7 (I) | 0 (R) | 18 (S) | 14 (S) | 13 (S) | 15 (S) | 20.5(S) | 19(S) | 14 (S) | 20(S) | 15 (S) | 19(S) | 18(S) |
| *Tetracyclin* | 24(S) | 26(S) | 30(S) | 20(S) | 36(S) | 32(S) | 36(S) | 15(S) | 12(S) | 34(S) | 10(S) | 36(S) | 12 (S) | 12(S) |
| ***Inhibitors of nucleic acid synthesis*** | | | | | | | | | | | | | | |
| *Ciprofloxacin* | 0 (R) | 0 (R) | 0 (R) | 16(S) | 18(S) | 28 (S) | 28 (S) | 26 (S) | 22 (S) | 18.5(S) | 24 (S) | 18.5(S) | 24 (S) | 24 (S) |
| *Levofloxacin* | 17(S) | 18(S) | 17(S) | 26(S) | 34(S) | 28(S) | 34(S) | 30(S) | 33(S) | 30(S) | 30(S) | 36(S) | 34(S) | 30(S) |
| *Nalidixic acid* | 0 (R) | 0 (R) | 0 (R) | 0 (R) | 0 (R) | 12(S) | 10 (S) | 0 (R) | 10(S) | 10 (S) | 16 (S) | 32 (S) | 8 (I) | 18 (S) |
| *Norfloxacin* | 0 (R) | 0 (R) | 0 (R) | 17(S) | 18(S) | 19(S) | 16(S) | 18(S) | 19(S) | 19(S) | 20(S) | 18(S) | 18(S) | 20(S) |
| *Rifampicin* | 32(S) | 31 (S) | 28(S) | 30 (S) | 30 (S) | 28 (S) | 32 (S) | 28 (S) | 30 (S) | 29 (S) | 23 (S) | 24 (S) | 31 (S) | 24 (S) |

* The definition of antibiotic-resistant (R), -susceptible (S) and –intermediate (I) of a given bacterial strain is based on the diameter of inhibition as compared to those reported by the EFSA (European Food Safety Authority, 2003 #10)

**Table S2.** Survival rates (%) * of the LAB strains exposed for 0 (T0), 1 (T1) and 3 (T 3) h to pepsin at pH 2 or pH 3 and of the LAB strains exposed for 0 (T0) and 4 (T4) h to pancreatin.

| | pepsin (pH=2) | | | pepsin (pH=3) | | | pancreatin | |
|---|---|---|---|---|---|---|---|---|
| | T0 | T1 | T3 | T0 | T1 | T3 | T0 | T4 |
| *L. paraplantarum* A1 | 89±8.9$^{a}$ | 79±5.0$^{a}$ | 77±1.1$^{b}$ | 65±1.4$^{A}$ | 59±2.3$^{A}$ | 58±2.0$^{A}$ | 71±2.6A | 46±3.3B |
| *L. plantarum* A2 | 81±1.6$^{a}$ | 75±1.1$^{a}$ | 68±2.5$^{b}$ | 68±1.6 $^{A}$ | 64±2.1$^{A}$ | 60±3.5$^{A}$ | 65±2.4A | 58±4.2A |
| *L. plantarum* A3 | 80±1.2$^{a}$ | 74±1.2$^{a}$ | 63±2.3$^{a}$ | 62 ±2.4$^{A}$ | 76±1.4$^{A}$ | 51 ±2.1$^{B}$ | 57±2.0A | 53±3.4A |
| *E. faecalis* A4 | 75±2.0 $^{a}$ | 67±2.7 $^{a}$ | 62±1.6 $^{a}$ | 87±5.2$^{A}$ | 69±1.2$^{A}$ | 66±2.1$^{A}$ | 71±2.1A | 87±6.1A |
| *E. faecalis* A5 | 49±2.9 $^{a}$ | 47±1.8 $^{a}$ | 41±2.6 $^{a}$ | 90±5.0$^{A}$ | 57±3.2$^{A}$ | 48±8.9$^{B}$ | 74±1.5A | 87±6.6A |
| *E. faecalis* A6 | 72±1.5 $^{a}$ | 56±0.0$^{a}$ | 45±1.1$^{b}$ | 90±6.3$^{A}$ | 77±1.4$^{A}$ | 50±2.7$^{C}$ | 75±1.6A | 86±7.0A |
| *E. faecalis* A7 | 65±2.7 $^{a}$ | 59±3.3 $^{a}$ | 60±0.8 $^{a}$ | 90±6.1$^{A}$ | 62±3.4$^{A}$ | 59±2.8$^{A}$ | 81±1.1A | 87±6.4A |
| *E. faecalis* A8 | 58±2.7 $^{a}$ | 52±2.1$^{b}$ | 36±2.1$^{c}$ | 92±5.9$^{A}$ | 80±2.5$^{A}$ | 79±1.1$^{A}$ | 79±1.6A | 88±6.8A |
| *E. faecalis* A9 | 56±3.1 $^{a}$ | 49±3.0$^{a}$ | 34±1.9$^{b}$ | 90±9.1$^{A}$ | 78±2.1$^{A}$ | 59±2.8$^{A}$ | 75±1.6A | 88±7.3A |
| *E. faecalis* A10 | 76±0.9 $^{a}$ | 75±1.5$^{a}$ | 46±2.9$^{b}$ | 87±9.5$^{A}$ | 73±1.8$^{A}$ | 60±3.7$^{A}$ | 83±1.0A | 88±7.6A |
| *E. faecalis* A11 | 63±2.8 $^{a}$ | 60±2.1$^{a}$ | 48±3.1 $^{a}$ | 84±9.4$^{A}$ | 50±2.6$^{B}$ | 48±1.8$^{C}$ | 76±1.2A | 89±7.0B |
| *E. faecalis* A12 | 63±1.5 $^{a}$ | 46±3.2 $^{a}$ | 36±1.1$^{b}$ | 83±1.3$^{A}$ | 60±1.5$^{B}$ | 61±1.4$^{A}$ | 85±6.8A | 89±6.9A |
| *E. faecalis* A13 | 36±2.1 $^{a}$ | 23±2.1$^{a}$ | 23±1.1$^{b}$ | 84±1.0$^{A}$ | 62 ±3.4$^{A}$ | 62 ±2.9$^{A}$ | 79±1.0A | 88±5.9B |
| *E. faecalis* A14 | 63±6.5 $^{a}$ | 58±1.9 $^{a}$ | 61±2.9 $^{a}$ | 92±5.6$^{A}$ | 67±2.2$^{B}$ | 69±1.6$^{C}$ | 87±6.7A | 90±5.6A |

* Mean values (±standard deviation) in the same row flanked by at least one common letter showed no significant (P>0.05) differences. Superscript letters refer to the treatment with pepsin at pH 2; superscript capital letters refer to the treatment with pepsin at pH 3; capital letters refer to the treatment with pancreatin.

**Table S3.** Antibacterial activity of LAB strains against target pathogenic bacteria.

| *LAB strain* | *S. aureus* | *S. epidermidis* | *M. luteus* | *E. coli* | *L. monocytogenes* | *P. aeruginosa* | *E. faecalis* | *S.* Typhimurium | *B. cereus* | *V. parahaemolyticus* |
|---|---|---|---|---|---|---|---|---|---|---|
| *A1* | ++ | ++ | ++ | ++ | + | - | ++ | ++ | + | + |
| *A2* | ++ | ++ | ++ | ++ | + | + | ++ | - | - | + |
| *A3* | ++ | ++ | ++ | ++ | + | + | + | - | - | - |
| *A4* | ++ | ++ | - | + | ++ | + | ++ | + | + | + |
| *A5* | ++ | ++ | + | + | + | ++ | ++ | + | + | + |
| *A6* | + | ++ | - | + | ++ | ++ | ++ | + | + | + |
| *A7* | ++ | ++ | + | + | ++ | ++ | ++ | + | + | + |
| *A8* | ++ | ++ | + | + | ++ | ++ | ++ | + | + | + |
| *A9* | ++ | ++ | + | + | ++ | ++ | ++ | + | + | + |
| *A10* | ++ | ++ | + | + | ++ | ++ | ++ | + | + | + |
| *A11* | ++ | + | - | + | ++ | + | + | + | + | + |
| *A12* | ++ | ++ | + | + | ++ | ++ | ++ | + | + | + |
| *A13* | ++ | + | + | + | ++ | ++ | + | + | - | + |
| *A14* | ++ | - | + | + | ++ | ++ | + | - | - | + |

**(-): no inhibition, Ø of inhibition zone <4 mm; (+): 4<Ø<10 mm; (++): Ø>10 mm**

**Table S4.** Anti-biofilm activity* of LAB strains against target pathogenic bacteria.

| ***LAB strain*** | ***S. aureus*** | ***S. epidermidis*** | ***M. luteus*** | ***E. coli*** | ***L. monocytogenes*** | ***P. aeruginosa*** | ***E. faecalis*** | ***S.* Typhimurium** | ***B. cereus*** | ***V. parahaemolyticus*** |
|---|---|---|---|---|---|---|---|---|---|---|
| **A1** | 63±3.0$^{ab}$ | 51±2.4$^{b}$ | 68±1.4$^{a}$ | 68 ±9.9$^{ab}$ | 76±8.4$^{a}$ | 65±3.2$^{b}$ | 43±3.6$^{c}$ | 46±3.6$^{b}$ | 86±5.1$^{a}$ | 88±2.7$^{a}$ |
| **A2** | 69±8.3$^{ab}$ | 53±8.6$^{ab}$ | 65±2.0$^{a}$ | 55±2.4$^{b}$ | 73±2.8$^{a}$ | 52±3.4$^{c}$ | 62±1.5$^{b}$ | 50±0.5$^{b}$ | 88±4.0$^{a}$ | 89±2.0$^{a}$ |
| **A3** | 56±2.6$^{b}$ | 47±1.8$^{b}$ | 71±2.3$^{a}$ | 45±19.0$^{b}$ | 70±1.1$^{a}$ | 80±1.3$^{a}$ | 50±4.0$^{bc}$ | 55±3.6$^{b}$ | 87±6.9$^{a}$ | 87±3.3$^{a}$ |
| **A4** | 69±7.4$^{ab}$ | 72±8.3$^{a}$ | 76±2.2$^{a}$ | 74±1.3$^{ab}$ | 46±1.3$^{b}$ | 71±3.4$^{ab}$ | 77±7.8$^{a}$ | 48±1.1$^{b}$ | 86 ±2.7$^{a}$ | 83±7.4$^{a}$ |
| **A5** | 74±0.0$^{a}$ | 78±2.1$^{a}$ | 81±1.8$^{a}$ | 76±6.2$^{a}$ | 42±1.2$^{b}$ | 82±1.1$^{a}$ | 66±2.0$^{a}$ | 49±4.9$^{b}$ | 83±4.0$^{a}$ | 84±1.0$^{a}$ |
| **A6** | 65±1.8$^{ab}$ | 50±8.1$^{b}$ | 75±3.1$^{a}$ | 75±1.8$^{ab}$ | 40±1.8$^{b}$ | 84±5.6 $^{a}$ | 51±3.2$^{bc}$ | 63±3.0$^{a}$ | 80±8.7$^{a}$ | 83±6.0$^{a}$ |
| **A7** | 63±5.4$^{ab}$ | 58±7.7$^{ab}$ | 78±2.1$^{a}$ | 87±1.7$^{a}$ | 51±1.7$^{b}$ | 74± 9.4$^{a}$ | 73±9.4$^{a}$ | 59±1.0$^{a}$ | 82±3.2$^{a}$ | 82±6.9$^{a}$ |
| **A8** | 54±1.6$^{ab}$ | 67±1.9$^{a}$ | 74±2.2$^{a}$ | 62±2.9$^{b}$ | 59±1.2$^{b}$ | 66±1.2$^{b}$ | 72±1.6$^{a}$ | 45±7.7$^{a}$ | 82±4.6$^{a}$ | 86±7.7$^{a}$ |
| **A9** | 64±2.4$^{ab}$ | 67±6.5$^{a}$ | 73±2.8$^{a}$ | 76±1.3$^{ab}$ | 63±8.3$^{a}$ | 74±6.1$^{a}$ | 72±3.4$^{a}$ | 58±5.5$^{a}$ | 84±4.7$^{a}$ | 85±9.6$^{a}$ |
| **A10** | 64±6.1$^{ab}$ | 55±1.6$^{b}$ | 69±3.0$^{a}$ | 71±2.5$^{b}$ | 38±9.8$^{b}$ | 69±1.3$^{b}$ | 71±4.2$^{a}$ | 71±1.4$^{a}$ | 83±2.4$^{a}$ | 81±9.6$^{a}$ |
| **A11** | 56±1.7$^{b}$ | 53±1.6$^{b}$ | 67±3.4$^{a}$ | 76±1.4$^{ab}$ | 31±1.1$^{c}$ | 72±5.0$^{ab}$ | 77±1.1$^{a}$ | 59±0.3$^{a}$ | 77±6.3$^{a}$ | 76±6.6$^{a}$ |
| **A12** | 76±6.5$^{a}$ | 68±2.2$^{a}$ | 73±2.4$^{a}$ | 73±1.1$^{b}$ | 53±4.2$^{ab}$ | 77±7.6$^{a}$ | 62± 1.7$^{b}$ | 59±2.3$^{a}$ | 78±8.7$^{a}$ | 83±4.9$^{a}$ |
| **A13** | 64±1.0$^{ab}$ | 75±2.0$^{a}$ | 72±2.7$^{a}$ | 75±1.7$^{ab}$ | 37±4.3$^{b}$ | 73±9.2$^{a}$ | 49±8.7$^{bc}$ | 74±1.0$^{a}$ | 76±9.8$^{a}$ | 81±2.9$^{a}$ |
| **A14** | 68±3.9$^{ab}$ | 64±5.0$^{a}$ | 76±2.4 $^{a}$ | 74±1.6$^{ab}$ | 35±9.4$^{b}$ | 67±3.3$^{ab}$ | 75±1.1$^{a}$ | 66±2.1$^{a}$ | 71±1.3$^{b}$ | 82±5.7$^{a}$ |

* Values in the same column with at least one common letter (a-c) showed no significant (P>0.05) differences.
